# Identification of resection plane for anatomical liver resection using ultrasonography-guided needle insertion

**DOI:** 10.3389/fsurg.2022.1035315

**Published:** 2023-01-23

**Authors:** Xin Zhang, Zhenhui Huang, Haiwu Lu, Xuewei Yang, Liangqi Cao, Zilong Wen, Qiang Zheng, Heping Peng, Ping Xue, Xiaofeng Jiang

**Affiliations:** Department of Hepato-Biliary-Pancreatic Surgery, The Second Affiliated Hospital of Guangzhou Medical University, Guangzhou, China

**Keywords:** hepatocellular carcinoma, ultrasonography, anatomical resection, hepatic vein, liver

## Abstract

**Purposes:**

To set up an easy-handled and precise delineation of resection plane for hepatic anatomical resection (AR).

**Methods:**

Cases of AR using ultrasonography-guided needle insertion to trace the target hepatic vein for delineation of resection planes [new technique (NT) group, *n* = 22] were retrospectively compared with those without implementation of this surgical technique [traditional technique (TT) group, *n* = 29] in terms of perioperative courses and surgical outcomes.

**Results:**

The target hepatic vein was successfully exposed in all patients of the NT group, compared with a success rate of 79.3% in the TT group (*P *< 0.05). The average operation time and intraoperative blood loss were 280 ± 32 min and 550 ± 65 ml, respectively, in the NT group. No blood transfusion was required in either group. The postoperative morbidities (bile leakage and peritoneal effusion) were similar between groups. No mortality within 90 days was observed.

**Conclusions:**

Ultrasonography-guided needle insertion is a convenient, safe and efficient surgical approach to define a resection plane for conducting AR.

## Introduction

Hepatectomy is the first-line therapeutic option for hepatocellular carcinoma (HCC) and hepatolithiasis (intrahepatic stones, IHS), which are endemic in the Asia-Pacific region (1, 2). Anatomical resection (AR) is widely accepted as superior to non-anatomical resection in terms of surgical outcomes and survival for patients with HCC, considering that portal tumor thrombosis and intrahepatic metastasis are responsible for recurrence and poor prognosis after curative hepatic resection (3, 4). Also, AR is more effective for bleeding control and parenchymal preservation, and thus more beneficial for reducing postoperative morbidity and mortality. Identifying the major vascular structures in relation to the affected liver tissue, determining the segments that must be resected, and precisely proceeding with resection following the anatomical margins are critical for effective AR with minimal blood loss and optimal preservation of liver function.

Hepatic veins are intrahepatic veins that drain blood into the inferior vena cava (IVC). AR is commonly based on liver sections and segments defined using Couinaud's classification, which divides the liver into eight segments based on three major hepatic veins (right, middle, and left) and the planes passing along the portal vein bifurcation. Identification of hepatic veins as an important landmark for segment delimitation is therefore essential for AR (5). The accumulated evidence has demonstrated the importance of careful review of hepatic vein anatomy and planning of AR accordingly (6, 7). Preoperative computed tomography (CT)/magnetic resonance imaging (MRI) and intraoperative ultrasonography have been valuable tools in recognizing these venous landmarks and delineating resection margins (8, 9). Surgical planning based on a three-dimensional (3D) model reconstructed from imaging has emerged as a promising approach to optimize the surgical procedure (10). However, despite the implementation of adjuvant imaging techniques, in clinical practice exact delineation of resection planes intraoperatively remains challenging. In the present study, we developed a simple technique for defining resection planes for AR in a precise manner. In this method, with the aid of intraoperative ultrasonography, a needle is inserted into the liver toward the target hepatic vein to create a resection plane for exposure of the hepatic vein. The feasibility and efficacy of this technique for creating resection planes for AR was assessed.

## Methods

This study was approved by the ethics committee of the Second Affiliated Hospital of Guangzhou Medical University. Informed consent was obtained from all patients. The use of ultrasonography-guided needle insertion to identify the resection plane in AR was initiated in January 2017, and as of July 2018, a total of 22 patients had undergone AR with this new technique (NT group). AR with this new technique was the preferred choice, unless the patient had a condition contraindicating the use of this approach, such as poor coagulation function or the absence of an appropriate puncture position. Another 29 patients who underwent liver resection without implementation of this new technique during the same period were used as reference cases [traditional technique (TT) group]. The surgeries in this study were all completed by Jiang's team, which was experienced and skillful in liver resection. The medical records of these patients were retrospectively reviewed, and the operation time, blood loss, transfusion rate, postoperative complications, and hospital stay were compared between the groups.

### Surgical procedures

**Figure 1:**
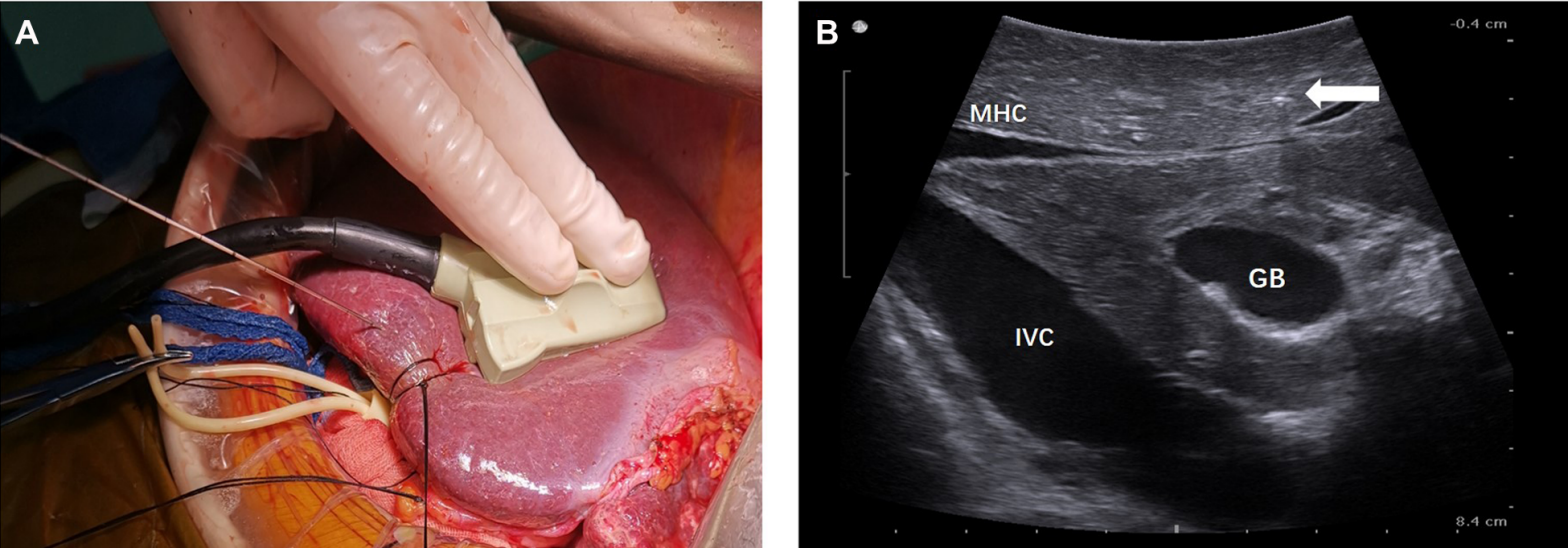
(**A**) Intraoperative view showing insertion of a 21-G needle into the liver toward MHV under ultrasonography guidance after ligation of the left portal vein and liver artery. (**B**) Intraoperative ultrasonography image showing the resection plane determined according to the MHV and the inserted needle (white arrow). The IVC may also be included in the resection plane. IVC, inferior vena cava; MHV, middle hepatic vein; GB, gallbladder.

**Figure 2:**
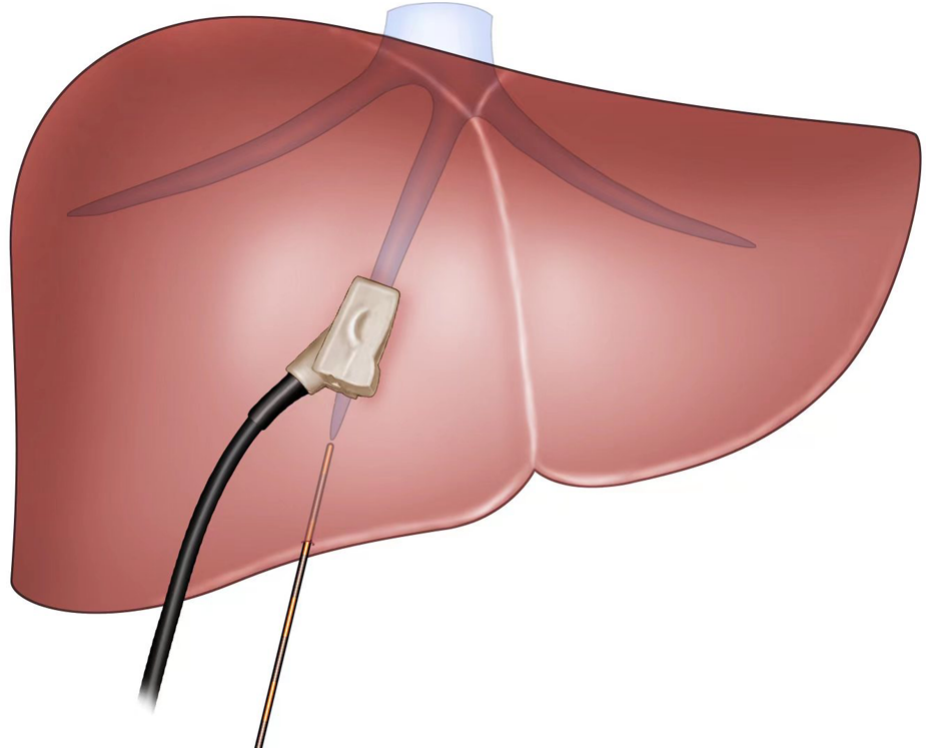
The view showing insertion of a 21-G needle into the liver toward MHV under ultrasonography guidance. MHV: middle hepatic vein.

**Figure 3:**
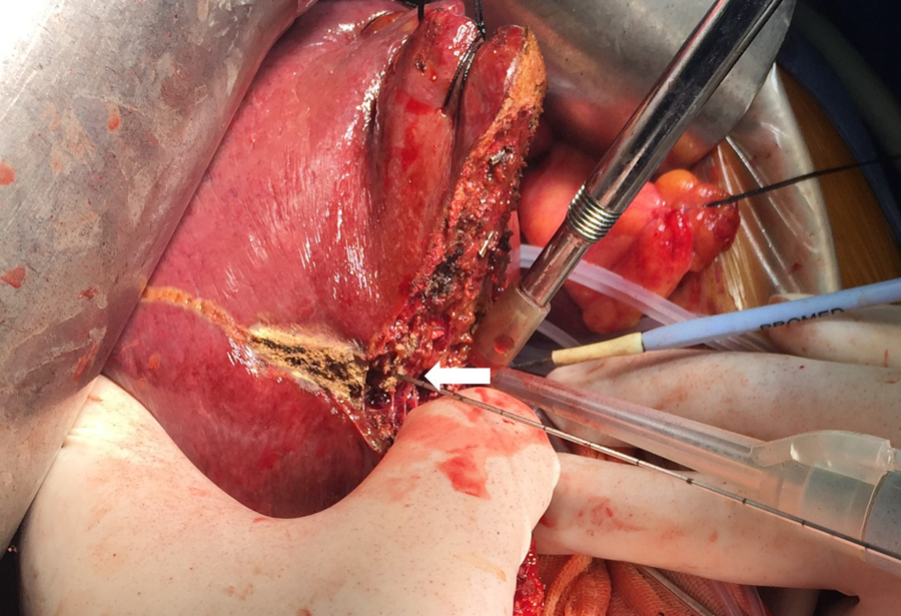
Intraoperative view showing parenchyma transection by a CUSA guided by the inserted needle (arrow) in a patient receiving left semi-hepatectomy.

The extent of AR was decided based on the size, number, and location of the lesions. For right or left hemi-hepatectomy, after mobilization of the liver according to the affected liver sections to be resected, selective ligation of portal veins and liver arteries was performed. For patients of the NT group, the resection plane was determined using the following steps. In step 1, intraoperative ultrasonography (BK Medical, Denmark) was performed to visualize the middle hepatic vein (MHV) and assess the appropriate position for needle insertion. In step 2, under ultrasonography guidance, a 21-G needle (Chiba, Japan) was inserted into the liver toward the MHV, as illustrated in Figure 1A. The insertion of the needle was confirmed under ultrasonography (Figures 1B, 2). A resection plane was defined by the inserted needle and the MHV (see Supplementary Video). Afterwards, parenchyma transection was initiated along the needle and continued until the MHV was reached with a cavitron ultrasonic surgical aspirator (CUSA, Integra, NJ, United States; Figure 3). In patients of the NT group receiving segmentectomy, to delineate the hepatic segment for AR, methylene blue was injected through the corresponding portal vein under ultrasonography guidance. Subsequently, a 21-G needle was inserted toward the corresponding hepatic vein and a resection plane was determined using the same approach described above.

In patients of the TT group, similar surgical techniques were used for AR, except for the method used to define the resection plane. Methylene blue was used to delineate the resection plane, and the resection line on the hepatic surface was marked with electrocautery under ultrasonography guidance.

### Statistical analysis

Continuous variables are expressed as mean ± standard deviation (SD) values, and categorized variables are expressed as percentages. Group differences were determined using Student's *t*-test for normally distributed variables and Mann–Whitney *U*-test if the variable did not follow a normal distribution. *χ*^2^ or Fisher's exact tests were used for comparison of categorical variables when appropriate. All statistical analyses were performed using SPSS 19.0 software (IBM SPSS Inc., IL, United States). *P* values <0.05 were considered statistically significant.

## Results

The primary outcome measure was the success rate of target hepatic vein exposure. The secondary outcome measures reflected the feasibility and safety of the approach which included the operation time, amount of blood loss, rate of blood transfusion, duration of hospital stay, postoperative morbidity and mortality. Table 1 summarizes the clinical, surgical, and outcome characteristics of the patients included in this study. No obvious differences were found between the NT and TT groups in terms of age, lesion types, size of tumor, number of tumor and types of resection.

**Table 1:** Baseline characteristics and surgical outcomes of patients.

	NT group (*n* = 22)	TT group (*n* = 29)	*P*
Age (years)	49 ± 6	53 ± 2	>0.05
Disease, *n* (%)			
HCC	12 (54.5)	16 (55.2)	>0.05
IHS	10 (45.5)	13 (44.8)	
Type of resection			>0.05
Hemihepatectomy	11	13	
Extended hemihepatectomy	2	2	
Sectionectomy	5	6	
Segmentectomy	4	8	
Resection plane, *n* (%)	22 (100)	23 (79.3)	<0.05*
Operation time (min)	280 ± 32	250 ± 15	>0.05
Blood loss (ml)	550 ± 65	600 ± 25	>0.05
Transfusion rate, *n*	0	0	>0.05
Hospital stay (day)	9.5 ± 1.5	10.5 ± 2	>0.05
Bile leakage, *n* (%)	1 (4.5)	2 (6.9)	>0.05
Peritoneal effusion, *n* (%)	2 (9.1)	2 (6.9)	>0.05

HCC, hepatocellular carcinoma; IHS, intrahepatic stones; TT, traditional technique; NT, new technique.

*Significant difference.

**Figure 4:**
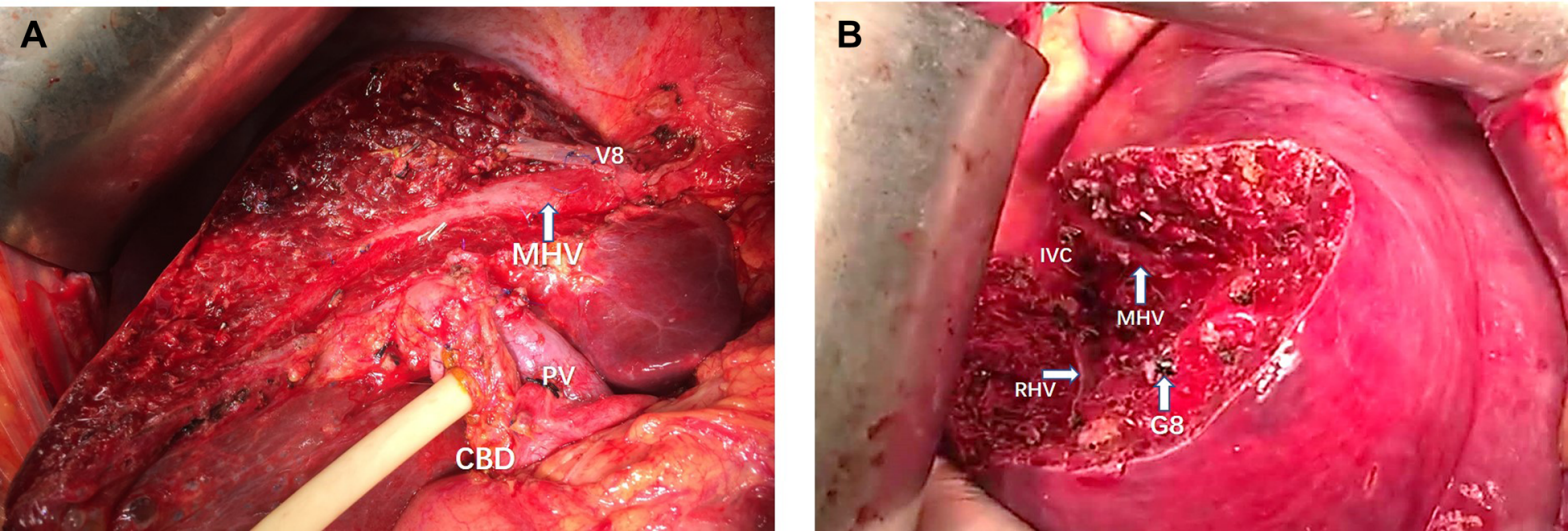
(**A**) Intraoperative view showing the MHV being exposed after left semi-hepatectomy for intrahepatic stones. (**B**) Intraoperative view showing the MHV, RHV, and IVC being exposed after segment 8 resection guided by the inserted needle. MHC, middle hepatic vein; PV, portal vein; CBD, common bile duct; RHV, right hepatic vein; IVC, inferior vena cava; V8, vein of segment 8; G8, Glisson's 8.

**Figure 5:**
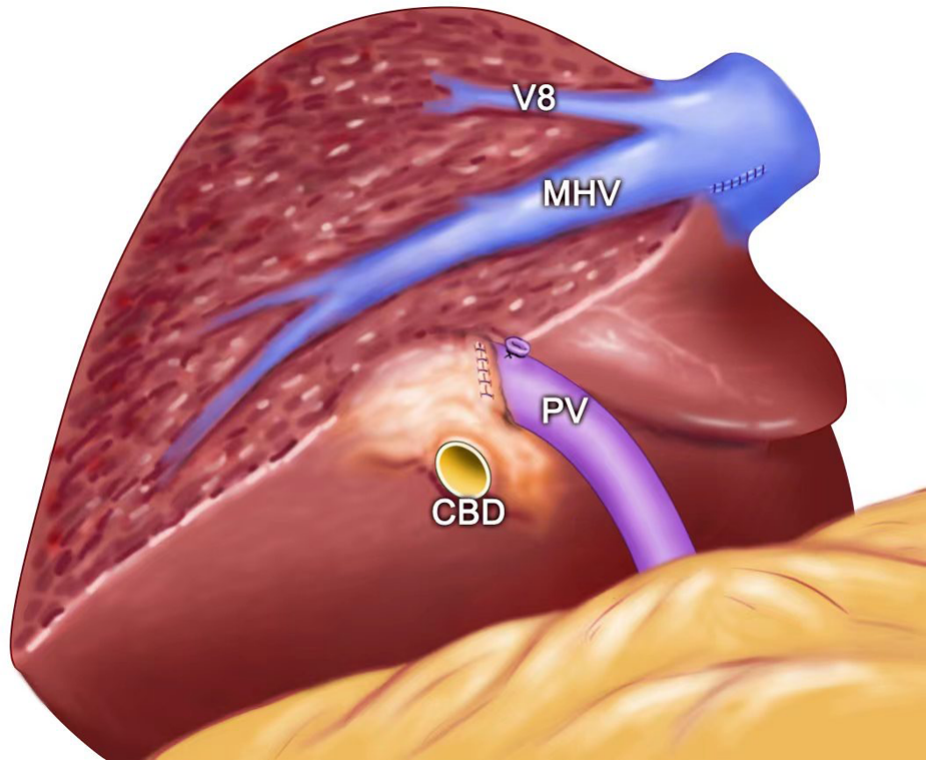
The view showing the MHV being exposed after left semi-hepatectomy for intrahepatic stones. MHC, middle hepatic vein; PV, portal vein; V8, vein of segment 8; CBD: common bile duct.

**Figure 6:**
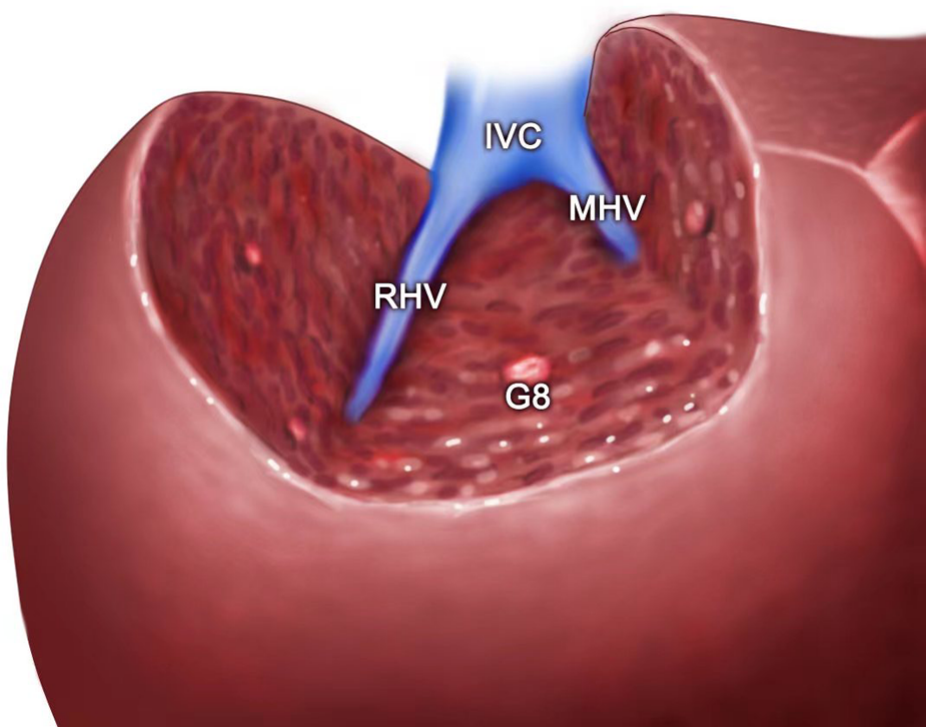
The view showing the MHV, RHV, and IVC being exposed after segment 8 resection guided by the inserted needle. MHC, middle hepatic vein; RHV, right hepatic vein; IVC, inferior vena cava; G8, Glisson's 8.

**Figure 7:**
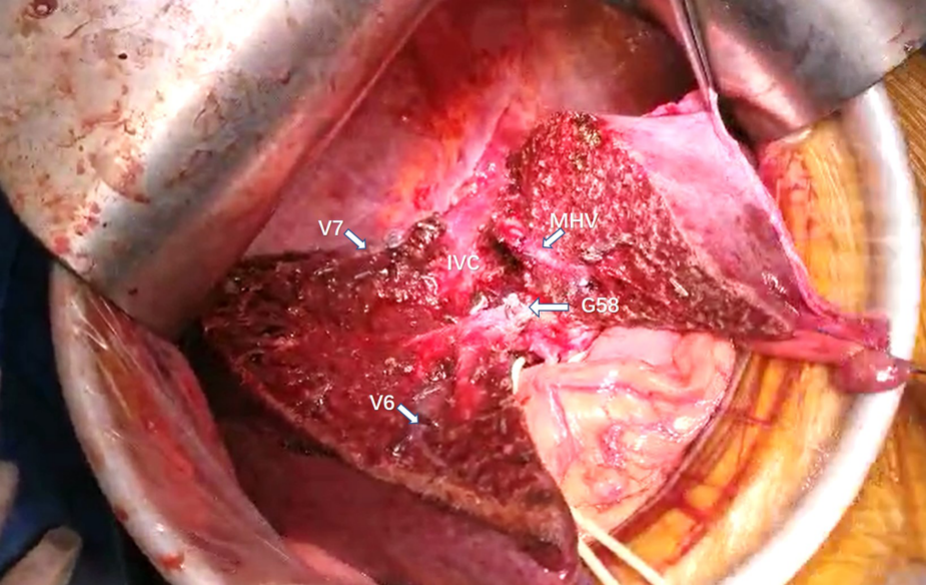
Intraoperative view showing the MHV, IVC, V6, and V7 being exposed after segment 5 and segment 8 resection for HCC. MHC, middle hepatic vein; V6, vein of segment 6; V7, vein of segment 7; IVC, inferior vena cava; G58, Glisson's 5 and 8.

**Figure 8:**
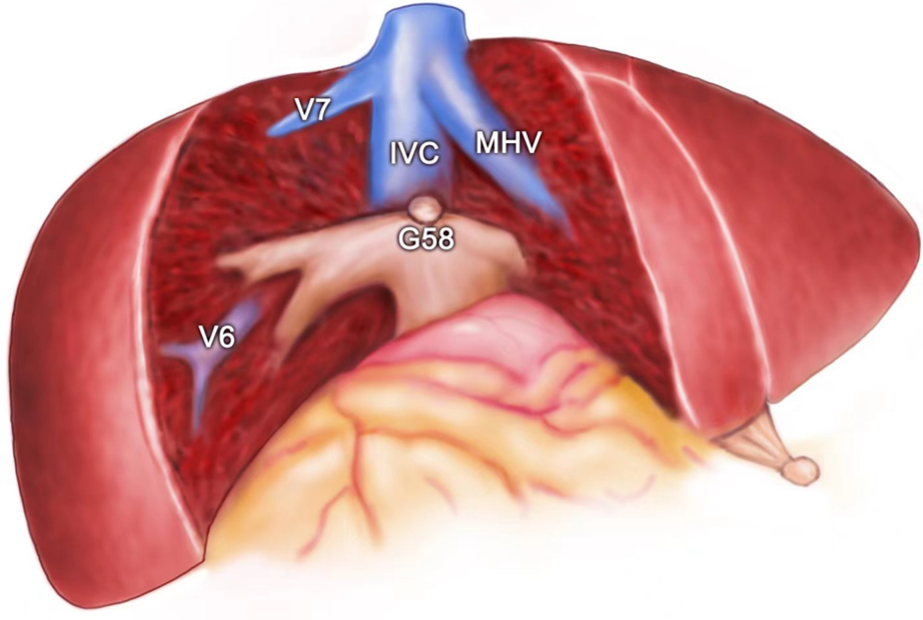
The view showing the MHV, IVC, V6, and V7 being exposed after segment 5 and segment 8 resection for HCC. MHC, middle hepatic vein; V6, vein of segment 6; V7, vein of segment 7; IVC, inferior vena cava; G58, Glisson's 5 and 8.

The attempt to define the resection plane for AR failed in one patient because of the occurrence of needle drop during the transection procedure, and this case was therefore not included in the NT group. After this event, we replaced the 20-cm-long needle with a 15-cm-long needle. Resection to expose the target hepatic veins as guided by the needle insertion method succeeded in all 22 patients of the NT group, while the success rate was significantly less at only 79.3% in the TT group (*P *< 0.05). An intraoperative view from a patient undergoing left hepatectomy for IHS in the NT group showed that the MHV was exposed after transection along the resection plane guided by the needle (Figures 4A, 5). The MHV, right hepatic vein (RHV), and IVC were exposed after segment 8 resection using the needle insertion method (Figures 4B, 6). In a patient with HCC in the NT group, the RHV was not identified under ultrasonography guidance. In this patient, resection planes guided by needles toward the vein of segment 6 (V6) and vein of segment 7 (V7) were created. The MHV, V6, and V7 were exposed after resection of segments 5 and 8 in this patent (Figures 7, 8).

The operation duration did not differ significantly between the NT and TT groups. The intraoperative blood loss volume was lower in the NT group than in the TT group, but the difference was not statistically significant. No blood transfusion was required in either group. The average duration of hospital stay of patients in the NT group was shorter but not significantly different compared with that for patients in the TT group.

The postoperative hospital morbidity rates were 9.1% in both the NT and TT groups (*P *> 0.05). No death was reported during the first 90 days after operation in either group.

## Discussion

AR is a technically challenging surgical procedure because of the potential risk of massive hemorrhage during surgical resection due to the complicated hepatic vascular anatomy. Over recent decades, significant technical advances have contributed to the reduction of perioperative hemorrhage, including better delineation of resection planes with the aid of preoperative and intraoperative imaging techniques, and more techniques available for inflow and outflow occlusion. In this study, patients received AR with these now considered standard-of-care surgical procedures, and the results were satisfactory for all patients in terms of perioperative hemorrhage, given that blood transfusion was not required for any patients who received AR.

Intrahepatic metastasis of HCC occurs mainly through the portal vein route. AR can not only eliminate the tumor but also remove the independent hepatic segment where the tumor is located as well as the portal vein branch within the hepatic segment, so as to completely remove the lesion and reduce the likelihood of tumor recurrence (7). AR can achieve the expected safe margin for tumor patients and can completely remove the diseased bile duct for patients with hepatolithiasis, thus reducing the incidence of postoperative biliary leakage. Studies have shown that compared with non-anatomical resection, AR can reduce the recurrence of tumors and improve survival (11–13). Therefore, AR should be considered the first choice for hepatectomy for HCC.

However, AR has not been accepted as a standard surgical treatment for HCC worldwide. One important reason is that the demarcation planes between liver segments are irregular, which is particularly evident in the right liver. It is not easy to accurately identify the demarcation plane during resection to execute true AR. According to Makuuchi (14), hepatic segmental/subsegmental resection must proceed precisely along the hepatic segmental boundary and fully expose the hepatic veins in order to be called AR. Active exposure of hepatic veins may avoid injury and reduce the risk of bleeding. Although satisfactory surface markers can be obtained by ligating the hepatic pedicle or injecting dye into the portal vein, the ischemia boundary within liver parenchyma is not obvious, and dye is prone to contaminate the contralateral side through cross-sectional leakage, thus blurring the boundary. As a consequence, the transection from the line marked on the surface of the liver may not be precisely along the direction towards the deep inside hepatic veins. In our study, not surprisingly, transection through the resection plane defined by the surface markers failed to expose the target hepatic vein in 20.7% of patients in the TT group. The hepatic vein, as the demarcation between liver lobes and segments, is a natural marker of the intrahepatic plane. Makuuchi chose to obtain the surface ischemia line by staining or regional block of hepatic pedicle, transect 1 cm down to find the subbranch of hepatic vein, and then separate the liver parenchyma down to the trunk of hepatic veins (14). However, when dissecting liver parenchyma, the subbranches of target hepatic vein encountered initially are relatively thin and vulnerable to injury. It may not only lead to bleeding and gas embolism, but could also lead to a loss of direction of the target hepatic vein, making it more difficult to expose the target hepatic vein. Moreover, in order to avoid damage to the branches of the target hepatic vein, the surgeon must be sufficiently meticulous, which may prolong the surgical procedure. To overcome this challenge, we developed a simple surgical procedure to define the resection plane for AR. Our results demonstrated the feasibility and efficacy of this approach based on a success rate of 100% for exposing the target hepatic veins.

In this study, we inserted a 21-G needle into the liver toward the direction of the target hepatic vein under the guidance of intraoperative ultrasonography. Based on the theorem that two intersecting lines determine one plane, the target hepatic vein and inserted needle make up a resection plane expected to expose the hepatic vein. A key step to ensure this resection plane passes through the hepatic vein is to have both the hepatic vein and inserted needle visualized as a line rather than a dot on the ultrasound screen. After the resection plane was established, we transected the liver parenchyma using a CUSA by following the inserted needle until the hepatic vein was reached. AR was carried out by tracking the hepatic vein until IVC exposure. These hepatic veins created the resection planes for AR. Our clinical experience demonstrated that compared with the traditional approach to identify the resection planes for AR, this method is straightforward and relatively easy to follow. In all patients in the NT group, all major hepatic veins were successfully exposed using this approach during the AR procedure. Notably, in one case in which the RHV was not visualized on the ultrasonography image, we easily established resection planes towards the V6 and V7 to achieve complete tumor resection. This demonstrated the flexibility of this method in the operative procedure in the case of anatomical variation.

The intraoperative blood loss and operation duration in the NT group were similar to those in the TT group, indicating that this ultrasonography-guided needle insertion method does not negatively affect the risk for hemorrhage or prolong the operation time. The postoperative hospital morbidity was similar between groups, and no mortality within 90 days of operation was recorded in either group, supporting the safety of this needle insertion procedure in AR for liver disease.

Another strength of this study is the use of the 21-G needle, which is widely used and not expensive in clinical settings. This method is therefore applicable and affordable in resource-poor regions. Indocyanine green (ICG) fluorescence-guided liver resection has emerged as a promising approach for AR by real-time illuminating anatomical landmarks of the liver (15, 16). However, this technique requires special equipment and reagents as well as complex surgical skills in portal vein puncture, which limits its application, especially in less developed regions.

To our best knowledge, this is the first report describing AR using an inserted needle to create resection planes for hepatic vein exposure under ultrasonography guidance. A previous study applied a needle insertion method for hepatic resection (17), but in that study, the needle was used to mark the distance from the tumor to the resection margin to guarantee adequate hepatic transection.

In conclusion, the ultrasonography-guided needle insertion method is a feasible and efficient procedure for identifying resection planes for AR. This technique provides a convenient and flexible approach for tracing hepatic veins during AR. Although our study is preliminary with its retrospective nature and small number of patients, we believe that the promising results observed in this study should lead to more researches. Further evaluation of the use of this technique in laparoscopic liver resection is expected.

## Data Availability

The original contributions presented in the study are included in the article/**Supplementary Material**, further inquiries can be directed to the corresponding author.
